# Defining the nociceptor transcriptome

**DOI:** 10.3389/fnmol.2014.00087

**Published:** 2014-11-11

**Authors:** Matthew Thakur, Megan Crow, Natalie Richards, Gareth I. J. Davey, Emma Levine, Jayne H. Kelleher, Chibeza C. Agley, Franziska Denk, Stephen D. R. Harridge, Stephen B. McMahon

**Affiliations:** ^1^Wolfson Centre for Age-Related Diseases, King's College LondonLondon, UK; ^2^Centre of Human and Aerospace Physiological Sciences, King's College LondonLondon, UK

**Keywords:** pain, nociceptors, nociception, somatosensation, dorsal root ganglion, RNA-sequencing, peripheral nervous system, regeneration

## Abstract

Unbiased “omics” techniques, such as next generation RNA-sequencing, can provide entirely novel insights into biological systems. However, cellular heterogeneity presents a significant barrier to analysis and interpretation of these datasets. The neurons of the dorsal root ganglia (DRG) are an important model for studies of neuronal injury, regeneration and pain. The majority of investigators utilize a dissociated preparation of whole ganglia when studying cellular and molecular function. We demonstrate that the standard methods for producing these preparations gives a 10%-neuronal mixture of cells, with the remainder of cells constituting satellite glia and other non-neuronal cell types. Using a novel application of magnetic purification, we consistently obtain over 95% pure, viable neurons from adult tissue, significantly enriched for small diameter nociceptors expressing the voltage gated ion channel Na_v_1.8. Using genome-wide RNA-sequencing we compare the currently used (10% neuronal) and pure (95% nociceptor) preparations and find 920 genes enriched. This gives an unprecedented insight into the molecular composition of small nociceptive neurons in the DRG, potentially altering the interpretation of previous studies performed at the tissue level, and indicating a number of novel markers of this widely-studied population of cells. We anticipate that the ease of use, affordability and speed of this technique will see it become widely adopted, delivering a greatly improved capacity to study the roles of nociceptors in health and disease.

## Introduction

Cell sorting techniques combined with transcriptional or epigenetic profiling provide a unique insight into how gene expression is regulated across different cell types. While cell type-specific profiling is now widely employed by researchers in the oncology and immunology fields (Hu et al., 2005; Heng et al., 2008), most researchers studying the nervous system continue to assess gene expression at the whole tissue level.

This is despite the fact that neural tissues contain a wide variety of different cell types such as epithelial cells, fibroblasts, glia and macrophages, as well as molecularly and functionally distinctive subsets of neurons. This heterogeneity significantly limits the usefulness of transcriptomic and epigenetic data generated to date. While sorting techniques, including fluorescence-activated cell sorting (FACS), have been applied to neural tissue, these often require transgenic animals and large amounts of cellular material in order to recover sufficient RNA, and are often better suited to neonate than adult neural tissue (Lobo et al., 2006).

In the peripheral nervous system, a commonly studied tissue is dorsal root ganglion (DRG). The peripheral nerve cell bodies contained in the DRG are important targets for investigators assessing sensory function, injury, regeneration and pain. Chronic pain is one of the leading causes of long term disability, estimated to affect one in five adults (Breivik et al., 2013). More than one in three patients suffering chronic pain report inadequate pain relief (Breivik et al., 2013)—yet many promising preclinical drug targets have not translated into effective treatments. One reason for this is the relatively poor understanding of the fundamental physiology and pathophysiology of pain-sensing nerve fibers (nociceptors, a subset of peripheral nerve cells) at the molecular level (Eijkelkamp et al., 2012).

We present a novel application of magnetic cell sorting (MACS) which generates a 95% pure nociceptor preparation from adult mouse DRG. This MACS preparation is enriched for small nociceptive neurons expressing the voltage gated sodium channel Na_v_1.8. Comparison of gene expression in mixed dissociated ganglion with the MACS purified nociceptor preparation reveals the transcriptional signature of nociceptors.

## Materials and methods

### Animals

Adult male C57Bl/6 mice weighing between 20 and 25 g were used for all experiments (Harlan, Bicester, UK). Animals were housed under standard conditions (12 h light/dark cycle, lights on between 7:00 a.m. and 7:00 p.m., free access to lab chow and water, groups of 4–8). All care of animals was in accordance with the United Kingdom Animals Scientific Procedures Act (1986). Na_v_1.8 TdTomato mice have been extensively characterized elsewhere (Shields et al., 2012).

### Tissue preparation

Mice were euthanized with a terminal dose of pentobarbital and decapitated. DRG were taken from all levels as previously described (Malin et al., 2007). For experiments on intact ganglia, DRG were snap frozen using liquid nitrogen, before being homogenized in Trizol (Invitrogen). For unsorted and MACS-sorted preparations, DRG were washed in F12 medium followed by incubation in dissociating enzymes (3 mg/mL dispase, 0.1% collagenase and 200 U/ml DNAse, all from Roche) for 30 min at 37°C. Dissociation enzymes were then removed and replaced with F12. Cells were triturated 5 times using a P1000 tip to disrupt the tissue. Cells in suspension were retained, and fresh F12 was added to the remaining undisrupted material. This process was repeated 5 times. Cells in suspension were filtered through a 40 μm filter and centrifuged for 5 min at 150 G. For unsorted preparations, cells were resuspended in Trizol at this point and snap frozen.

### MACS-sorting of dissociated dorsal root ganglia

Dissociated DRG neurons were washed in Dulbeccos PBS then resuspended in 120 μl MACS buffer (Miltenyi autoMACS HBSS-based washing solution with 0.5% bovine serum albumin). 30 μl of biotinylated non-neuronal antibody cocktail was added (Miltenyi MACS Neuron Isolation Kit) and cells incubated at 4°C for 5 min. After washing in MACS buffer, cells were resuspended in 120 μl MACS buffer with 30 μl anti-biotin microbeads (Miltenyi MACS Neuron Isolation Kit) and incubated at 4°C for 10 min. Cells were then run through a LD exclusion column placed in a QuadroMACS separator (Miltenyi Biotech), which retained all microbead-conjugated, non-neuronal cells so that only neurons were eluted. For MACS-sorted preparations, eluted pure neurons were then centrifuged, resuspended in Trizol and snap frozen.

### Immunocytochemistry

For characterization, unsorted preparations were plated on poly-L-lysine/laminin coated coverslips. Sorted cells were plated on poly-L-lysine coverslips incubated with Matrigel (BD Biosciences) diluted 1:10 in F12. After ~18 h *in vitro*, cells were fixed in 2% PFA at 37°C for 10 min. Cells were then blocked in 10% Normal Donkey Serum in PBS with 0.2% Triton-X for 30 min at room temperature, followed by a 30 min RT incubation in primary antibody. Cells were washed in PBS and incubated in secondary antibody at room temperature for 30 min, followed by further washing and coverslipping with Vectashield mounting medium (Vector Labs). Primary antibodies used were: mouse β-III tubulin (Promega, G712A), rabbit glutamine synthetase (Abcam, ab498371), mouse CGRP (Abcam, ab81887). Secondaries were all Alexa 488 or 546 (Life Technologies). Fluorescence was visualized with fixed exposure times using a Zeiss Axioplan 2 microscope and analyzed using ImageJ software and Graphpad Prism. Positive cells were defined as cells with an intensity >30% that of the mean top 10 most intense cells in the image being analyzed.

### Flow cytometry of dissociated neuronal preparations

Cells were prepared for cytometry as described above, with two variations. Cells were prepared in cytometry buffer instead of medium (Hank's Buffered Saline Solution with 1 M HEPES and 0.5 M EDTA). Cells were prepared from mice expressing GFP in Advillin-positive cells, i.e., all DRG neurons (Advillin-GFP mouse obtained from Gensat). The pan-nuclear marker DRAQ5 (Biostatus, UK) was then added at 1:10,000. Unsorted and MACS sorted samples were analyzed using an LSR Fortessa. The number of GFP-positive events were quantified as a percentage of all DRAQ5-positive events to assay the proportion of neuronal to non-neuronal cells.

### RNA extraction and library preparation for sequencing

RNA was extracted from intact, unsorted and MACS-sorted samples using the RNeasy micro kit (Qiagen). Each sample (each *n*) comprised material from 1, 2 or 8 mice in the intact, unsorted and MACS-sorted groups, respectively. RNA integrity was assessed on an Agilent2100 Bioanalyzer (Agilent, Santa Clara, CA) and the RNA integrity number of each sample was >8. PolyA-selected RNA was used for complimentary DNA library preparation using the NEBNext Ultra direction RNA Library Prep Kit according to manufacturer's instructions (NEB, Ipswich, MA). 100 bp reads were sequenced on the Illumina HiSeq2000/2500 platform in replicate flow cells.

### Data analyses

Quality control, alignment and differential expression analyses were performed on the Galaxy server (Giardine et al., 2005; Blankenberg et al., 2010; Goecks et al., 2010). Cortical neuron fastQ files were uploaded to Galaxy directly from the European Nucleotide Archive (http://www.ebi.ac.uk/ena/data/view/SRP033200, sample accessions SAMN02415125, SAMN02415124). Briefly, fastQ files were uploaded, assessed for sequencing quality using fastQC (http://www.bioinformatics.babraham.ac.uk/projects/fastqc) and Picard Alignment Summary Metrics (http://picard.sourceforge.net). Base calls were of high quality across the full-length of the reads and trimming was not required. FastQ files were aligned to the UCSC mm10 build of the mouse genome using TopHat2 (Kim et al., 2013), with the multiple alignment parameter (-g) set to two. Aligned bam files from separate lanes were combined using the Merge Bam tool from SamTools (Li et al., 2009), then fragments per kilobase per million (FPKM) estimates were obtained using Cufflinks version 2.1.1 (Trapnell et al., 2012). Genes were considered to be expressed if more than three samples within a group had an FPKM value greater than 5.

Differential expression analyses were performed using CuffDiff version 2.1.1 (Trapnell et al., 2012). Genes were considered to be differentially expressed if they had a *q* < 0.05, log2-fold change < −0.5 or >0.5, and mean FPKM > 5 in the group with higher expression. Following differential expression analysis of MACS sorted samples it became clear that these were contaminated with red blood cells (e.g., they had high levels of canonical blood genes, such as *Hbb-bs*). To account for this, all genes that were found to be both significantly upregulated in MACS samples and had a median log2 RPKM > 3 in a recently published mouse erythrocyte transcriptome study (An et al., 2014) were removed prior to subsequent analysis (totaling 308 blood genes).

Principal component analysis, density and scatter plots were created using the cummeRbund package in R (version 3.1.0). Hierarchical clustering and heat maps were generated using MultiExperiment Viewer (MeV) (Saeed et al., 2003). DAVID (Da Wei Huang and Lempicki, 2008; Huang et al., 2009), Panther (Mi et al., 2013), and STRING (Jensen et al., 2009) were used for gene functional annotation and network analyses.

Datasets are available via GEO accession number GSE62424.

## Results

### MACS-sorted pure neuronal preparations are enriched for nociceptors

**Figure 1:**
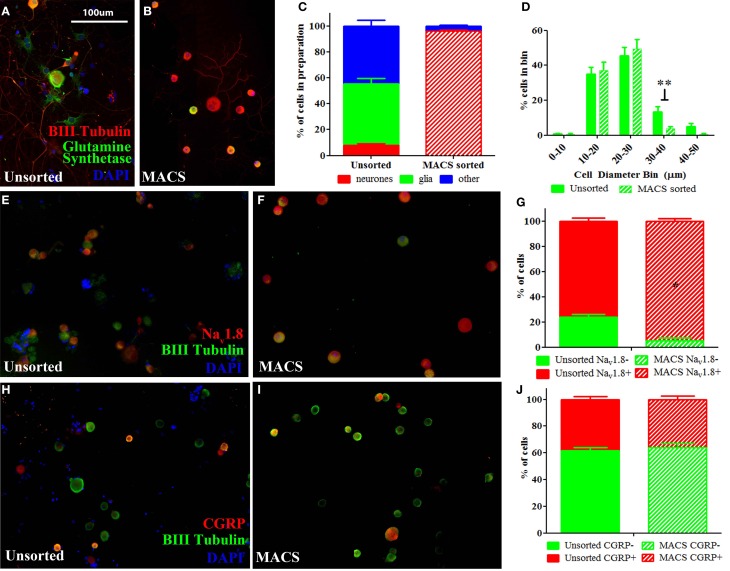
**Characterisation of MACS-sorted DRG neuronal preparation. (A,B)** Immunohistochemistry visualizing neuronal (β-III tubulin), satellite glial (glutamine synthetase) or other non-neuronal (DAPI) cells in unsorted and MACS-sorted neuronal preparations. **(C)** Quantification of neuronal, glial and other non-neuronal cells shows that MACS-sorting generates >95% pure neuronal preparation (*n* = 8 per condition). **(D)** Cell size distribution of neuronal cells in unsorted and MACS-sorted preparations shows that the MACS-sorted preparation is enriched for small cells <30 μm in diameter (*n* = 8 per condition. ^**^ = unpaired *t*-test *p* < 0.01). **(E,F)** Immunohistochemistry of unsorted and MACS-sorted preparations generated from Na_v_1.8 TdTomato reporter mice. **(G)** Percentage of cells positive for Na_v_1.8 is significantly greater following MACS sorting (*n* = 4 per condition, Fisher's exact test, ^*^p < 0.05). **(H,I)** Immunohistochemistry visualizing the neuropeptide calcitonin gene related peptide (CGRP) in unsorted and MACS-sorted preparations. **(J)** The percentage of cells expressing CGRP is unchanged in MACS-sorted preparations (*n* = 4, Fisher's exact test *p* = 0.229).

We used a novel application of magnetic cell sorting (MACS) to obtain a 95% pure peripheral nerve cell body preparation from adult DRG. Quantification of traditionally prepared, unsorted dissociated DRG cultures stained for the neuronal marker β-III Tubulin, the satellite glial cell marker glutamine synthetase (GS) and the nuclear marker DAPI demonstrate that neuronal cells constitute a mean of 9% of adherent, nucleated cells present in these preparations one day after dissociation (Figures 1A,C). In contrast, a DRG preparation subjected to MACS contains more than 95% neuronal nuclei (Figures 1B,C). Cell size distribution indicates that magnetic sorting generates a preparation enriched for cells < 30 μm in diameter (nociceptors) (Figure 1D).

Flow cytometry analysis of acutely dissociated DRG preparations confirms that neurons constitute 9.8% of nucleated events in the acute preparation (Supplementary Figure 1). This indicates that the relatively large proportion of non-neuronal cells detected after 1 day in culture do not simply reflect proliferating cells, but is an accurate reflection of the cellular makeup of the acute preparation. Note that while we observed that neurons only constituted ~10% of cells in acutely dissociated preparations of DRG, this is likely an underestimate of the total proportion of neurons in the intact DRG as the dissociation process will cause some cells, such as larger DRG neurons, to lyse.

We next performed MACS sorting on dissociated DRG from transgenic animals carrying the TdTomato reporter in cells expressing the voltage-gated sodium channel, Na_v_1.8 (Figures 1E,F). This confirmed that the MACS sorting process generates a cell preparation enriched for nociceptors. In agreement with previous studies (Shields et al., 2012), Na_v_1.8+ cells make up only 76% of dissociated unsorted neurons, yet constitute 95% of the MACS sorted preparation (Figure 1G).

The proportion of cells expressing the neuropeptide calcitonin gene-related peptide (CGRP), which defined a subset of nociceptors, is similar in unsorted (Figure 1H, 38%) and sorted preparations (Figure 1I, 36%), indicating that the MACS sorting process enriches cells according to size but introduces no bias in neurochemical subtype (Figure 1J).

### RNA-sequencing of intact, dissociated and MACS-sorted preparations identifies the nociceptor transcriptome

**Figure 2:**
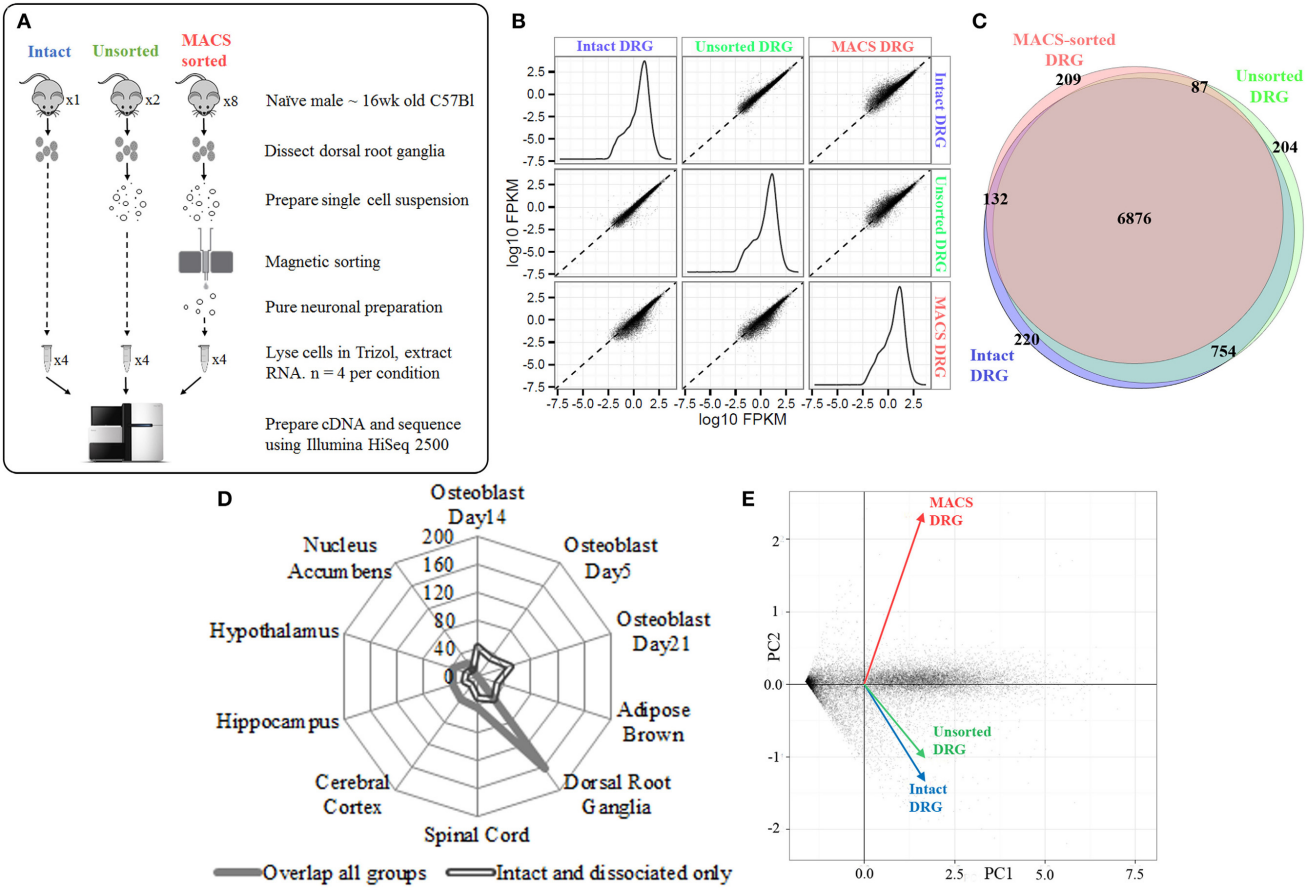
**Next-generation sequencing of intact, unsorted and MACS-sorted DRG neuronal preparations. (A)** Schematic illustrating study design for sequencing. Four replicate samples were sequenced in each group. **(B)** Graphs illustrate the distribution of FPKM values for all genes in each experimental group. Similar distributions were obtained for all groups, indicating that all groups have comparable coverage of genes across a range of expression levels. Scatterplots compare group-to-group log10 FPKM values. Divergence from the fit line indicates dissimilar gene expression patterns. **(C)** Venn diagram illustrating detection of 7987 genes in intact DRG, 7929 genes in unsorted samples, and 7309 genes in MACS-sorted samples. The vast majority of genes (6876, 81%) were expressed in all three groups. **(D)** Cell-type enrichment analysis suggests that genes common to all three groups are indicative of DRG and other neuronal cell types (filled line), while genes found only in the intact and dissociated groups were non-neuronal and most closely resembled osteoblast and brown adipose tissue (hollow line). **(E)** Principal component analysis (PCA) performed on all genes. Intact DRG and dissociated, unsorted samples cluster closely together, while MACS-sorted samples cluster separately. Plotted values are -log10 Bonferroni-corrected *p*-values.

We next extracted high-quality RNA from either whole, intact DRG, acutely dissociated, unsorted DRG or MACS-sorted DRG for use in RNA-sequencing (study design illustrated in schematic Figure 2A). The sequencing data was used to identify unbiased gene expression profiles across the entire transcriptome. Sequencing was performed on polyA+ messenger RNA of four biological replicates for each condition at a depth of ~18 million reads per sample. Sequencing data were subject to quality control analyses prior to alignment with TopHat2. A high-confidence transcriptome was generated using Cufflinks in Galaxy, and differences in gene expression levels were analyzed using CuffDiff. Only annotated genes were analyzed.

We initially determined how many genes were expressed in all three conditions. We detected 7987 genes in intact DRG, 7929 genes in dissociated, unsorted samples and 7309 genes in MACS-sorted samples, corresponding to ~30% of known murine genes (Figure 2B). The vast majority of these genes (6876, 81%) were expressed in all three groups (Figure 2C).

Cell-type enrichment analysis performed using the CTen platform (Shoemaker et al., 2012) accurately identified the overlapping genes as DRG and other neuronal cell types (Figure 2D). In contrast, genes found only in the intact and dissociated groups were not identified as neuronal, confirming the ability of the MACS sorting process to exclude non-neuronal cells.

Principal component analysis (PCA) was performed on all genes. Intact DRG and dissociated, unsorted samples clustered closely together, while MACS-sorted samples clustered separately (Figure 2E). Pairwise comparison of replicates within each group revealed a high degree of correlation (r2 = 0.81–0.87), indicating the reproducibility of the preparation methods.

### The MACS-sorted cell transcriptome identifies novel nociceptor-enriched ion channel genes

**Figure 3:**
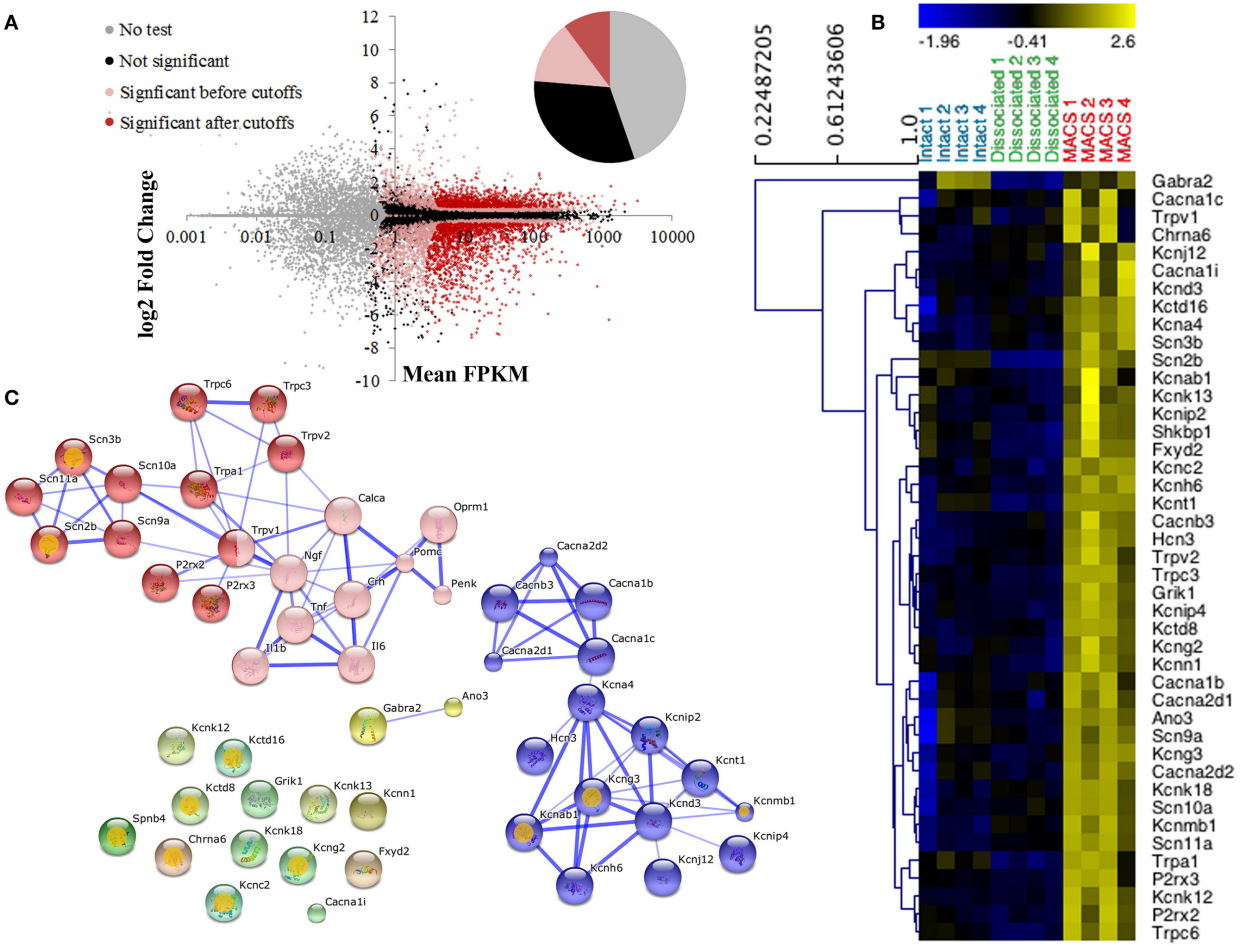
**Differential expression profiling of unsorted and MACS-sorted DRG cultures. (A)** Cuffdiff was used for differential expression testing between unsorted and MACS-sorted samples. Individual points represent genes. Of 23,991 annotated genes, 10,724 (44.7%) were not tested due to their low expression levels. Of the remaining genes, 7579 (31.6%) were not significantly differentially expressed and 5688 (23.7%) were. Of these, 2443 (10.2% of total) passed stringent fold change and expression criteria and were used for downstream analysis. **(B)** Heat map of ion channel expression in intact DRG, unsorted and MACS-sorted cultures. Genes are hierarchically clustered using average linkage clustering and expression is normalized by gene. Ion channels were identified by functional annotation enrichment analysis of MACS genes as they were found to be the most enriched gene class (GO term “ion channel activity,” *p* = 1.89E-10, FDR = 2.80E-07) **(C)** Network analysis of ion channel genes and pain-associated genes. Pink nodes represent pain-associated genes from the pain interactome (Jamieson et al., 2014); orange spots indicate genes with no known pain association; thickness of the edge represents the confidence of the interaction. Unbiased MCL clustering revealed two primary sub-clusters of ion channel genes, the majority of which have been previously studied in the context of pain. Many of the ion channels that do not group into these clusters are novel targets in the context of pain.

Nociceptor genes were identified by comparing MACS-sorted and unsorted DRG preparations using the CuffDiff algorithm. A total of 2392 differentially expressed genes were discovered, of which 920 (38.5%) were enriched in the MACS-sorted samples and 1473 (61.5%) were depleted (Figure 3A).

Consistent with the *in vitro* characterization, genes enriched in the MACS-sorted samples include many known small neuronal markers such as GDNF responsive receptor tyrosine kinase, *Ret* (log2-fold change = 0.79, *q* = 0.0002) and neurofilament peripherin (*Prph* log2-fold change = 0.39, *q* = 0.011). Depleted genes include the large neuronal marker parvalbumin (log2-fold change = −3.34, *q* = 0.0002) and the glial cell glutamate transporter GLAST-1 (*Slc1a3*, log2-fold change = −6.99, *q* = 0.0044). The genes enriched in MACS-sorted neurons represent all genes expressed more abundantly in nociceptors than in other DRG neurons and non-neuronal cells. To determine whether these genes may also be nociceptor-specific, we performed an *in silico* analysis of MACS transcriptional data against global gene expression data from pure cortical neurons (Zhang et al., 2014). 6140 genes were found to be differentially expressed between the two datasets, of which 3190 (52%) were enriched in cortical neurons and 2950 (48%) were enriched in nociceptors (described in Supplementary Figure 2 and Supplementary Table 2). Of the 920 genes most enriched in the MACS-sorted preparation compared to unsorted DRG, 548 (68%) were also significantly enriched compared to cortical neurons. This suggests that these genes are not only nociceptor-enriched, but may in fact be nociceptor-specific.

Table 1 below shows highlighted results. All MACS vs. DRG values are accessible in Supplementary Table 1 and MACS vs. Cortical Neurons may be found in Supplementary Table 2.

**Table 1:** **Genes with known roles in pain and somatosensation are enriched in MACS-sorted nociceptors compared to unsorted DRG (see also Supplementary Table 1)**.

**Gene name**	**Full gene description**	**Fold change MACS vs. dissociated DRG**	**Fold change MACS vs. cortical neurons**
*Calcb*	Calcitonin-related polypeptide beta	1.46	487.22
*Mrgpra2b*	MAS-related GPR, member A2B	1.87	inf
*Mrgpra3*	MAS-related GPR, member A3	4.42	inf
*Mrgprb4*	MAS-related GPR, member B4	1.86	inf
*Mrgprb5*	MAS-related GPR, member B5	3.25	inf
*Mrgprd*	MAS-related GPR, member D	2.01	inf
*Mrgpre*	MAS-related GPR, member E	1.48	5.63
*Mrgprx1*	MAS-related GPR, member X1	3.19	inf
*P2rx2*	Purinoreceptor 2	3.36	4.95
*P2rx3*	Purinoreceptor 3	1.90	695.23
*P2ry1*	Purinergic receptor P2Y, G-protein coupled, 1	2.51	7.37
*Prph*	Peripherin	1.80	1189.14
*Ret*	RET (GDNF receptor)	1.74	190.11
*Scn10a*	Na_v_1.8	1.71	8010.07
*Scn11a*	Na_v_1.9	2.14	3084.88
*Scn9a*	Na_v_1.7	1.47	38.76
*Sst*	Somatostatin	2.41	0.02
*Tac1*	Protachykinin-1 (Substance P precursor)	1.53	24.97
*Trpa1*	TRPA1	1.83	176.05
*Trpv1*	TRPV1	1.47	50.21
*Trpv2*	TRPV2	1.56	5.44

Many of these genes are also enriched in MACS-sorted nociceptors compared to cortical neurons, indicating that these genes are not only nociceptor-enriched, but may in fact be nociceptor-specific (see also Supplementary Figure 2 and **Table 2**).

Functional annotation clustering using DAVID indicates that genes enriched in MACS-sorted preparations possess GO terms related to ion channel activity and ion binding (false discovery rate corrected *p*-value range 6.55e-4–2.8e-7; Supplementary Table 3), and 43 genes related to ion channel activity were identified for downstream analysis (Figure 3B).

Jamieson et al. (2014) have recently described the pain interactome, a pain-specific protein-protein interaction network (Jamieson et al., 2014). Using the key nodes of the pain interactome alongside the 43 ion channel activity genes in an unbiased network analysis we discovered two major gene clusters (Figure 3C). The largest cluster contained the known pain-related genes from the pain interactome as well as various transient receptor potential channel members, purinoreceptors and voltage-gated sodium channel subunits, the majority of which have known functions in somatosensation or nociception. The second cluster primarily contained calcium and potassium channel subunits, including the calcium channel subunit *Cacna2d1* (alpha-2-delta-1) which is the major target of the neuropathic pain drug, gabapentin (Bauer et al., 2009). The remaining ion channel activity-related genes did not form clusters as they had no known interactions with either the pain-related genes or the calcium and potassium channel subunits, therefore representing a novel subset of nociceptor-enriched ion channel genes, the majority of which have not been previously linked to pain.

### Differential splicing of the TrkB receptor in neurons and non-neuronal cells

**Figure 4:**
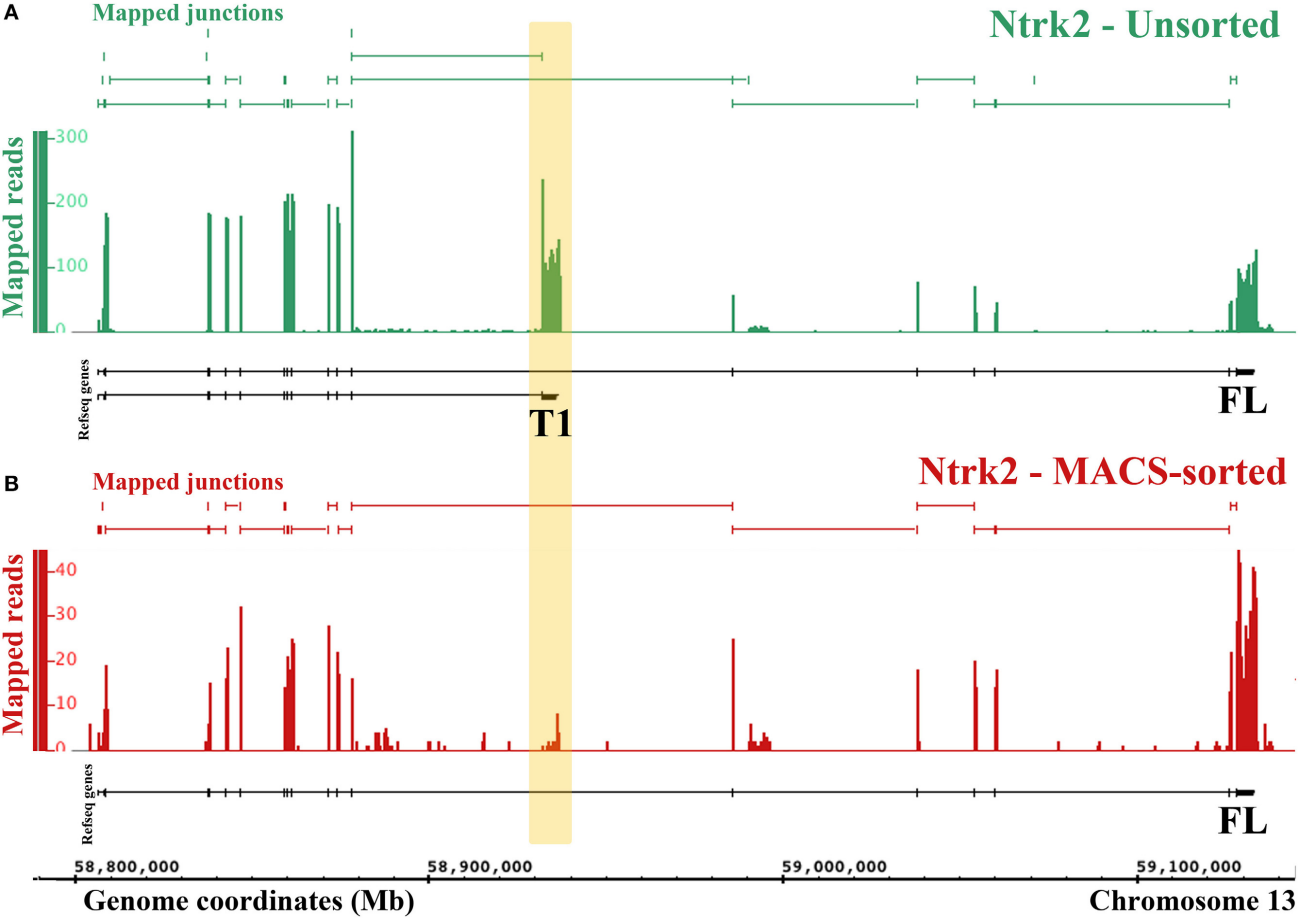
**RNA-Seq reveals differential expression of Ntrk2 isoforms in MACS-sorted cultures**. Figure generated by the Integrated Genome Browser using mapped reads from one MACS-sorted **(B)** and one unsorted sample **(A)**. Both Ntrk2 isoforms are depleted from MACS-sorted cultures, though some message remains. Interestingly, the junction between exons (16 and 17) is not detected in the MACS-sorted cultures, implying that this isoform is not expressed in nociceptors.

One major advantage of RNA-Sequencing over microarrays is the ability to measure isoform-level differences between samples. Using Cuffdiff we identified 2444 transcripts that were differentially expressed between MACS-sorted samples and unsorted DRG samples, of which 1473 were depleted and 971 were enriched in nociceptors, as well as 99 genes with alternate promoter use and 63 genes that were differentially spliced (Supplementary Table 4). Interestingly, one of the genes that was significantly differentially expressed at the transcript level was the brain-derived nerve growth factor (BDNF) receptor, *Ntrk2* (TrkB). There are two isoforms of mouse *Ntrk2*: one (NM_001025074) encodes the full-length receptor and the other (NM_008745) encodes a truncated version. Although both isoforms were depleted from MACS cultures, the truncated form was downregulated to a much greater extent (log2-fold change = −2.9, = −6.9, Figure 4) and the message was virtually absent (mean FPKM = 0.23, s.e.m. = 0.12, Figure 4 and Supplementary Table 4). This was similar to what was observed for known non-neuronal genes in the MACS-sorted samples, for example the voltage gated potassium channel Kir4.1 (*Kcnj10*, FPKM = 0.15, Supplementary Table 1) and the glutamate transporter GLAST-1 (*Slc1a3* FPKM = 0.04, Supplementary Table 1). This suggests that, in contrast to CNS tissues where the truncated form is expressed in both neuronal and non-neuronal cells, in DRG truncated TrkB is primarily expressed in non-neuronal cells while neurons only express the full length isoform.

Isoform-specific expression analyses were also performed against cortical neurons. These results may be accessed in Supplementary Table 5.

### MACS-sorting identifies novel transcription factors present in nociceptors

Transcriptional regulation is an important mechanism for chronic pain (Perkins et al., 2014). Functional annotation analysis of nociceptor-enriched genes identified 70 genes associated with nucleic acid binding and transcription factor activity (the top 15 are presented in Table 2, below, with the full list in Supplementary Figure 3). This list includes *Tlx3* and *Pou4f1*, genes that are known to be critical for sensory neuron development (Lopes et al., 2012; Zou et al., 2012) as well as 49 genes whose role in pain and somatosensation is not yet known. Unbiased network analysis indicates that among these newly identified nociceptor transcription factors, *Klf5*, *Med10*, *Myc*, and *Stat6* are predicted to interact with key nodes of the pain interactome (Supplementary Figure 4; Jamieson et al., 2014).

**Table 2:** **The most highly enriched genes with nucleic acid binding functions present in nociceptors**.

**Gene symbol**	**Full gene name**	**FPKM unsorted**	**FPKM MACS**	**log2-fold change**
*Bhlha9*	Basic helix-loop-helix family member a9	5.45	26.06	2.26
*Klf5*	Krueppel-like factor 5	14.20	43.55	1.62
*Ldb2*	Lim domain binding 2	47.25	119.58	1.34
***Sox11***	SRY-box 11	2.30	5.55	1.27
*Pou4f2*	POU domain class 4 transcription factor 2/Brn3b	7.57	18.23	1.27
***Pdlim1***	PDZ and LIM domain protein 1	6.50	15.58	1.26
*Zbtb48*	Zinc finger and BTB containing 48	2.24	5.28	1.23
*Atf5*	Activating transcription factor 5	32.35	75.84	1.23
***Isl2***	Islet 2	81.72	190.99	1.22
***Tlx3***	T-cell leukemia homeobox 3	38.59	86.16	1.16
***Myt1***	Myelin transcription factor 1	15.02	33.49	1.16
*Grhl3*	Grainyhead-like 3	3.73	8.26	1.15
*Casz1*	Castor zinc-finger 1	14.45	31.99	1.15
*Prdm12*	PR domain containing 12	64.85	142.95	1.14
*Aff2*	AF4/FMR2 family member 2	2.92	6.42	1.13

Genes already known to have a role in these cells are highlighted in bold.

Importantly, comparison of intact DRG vs. dissociated DRG samples indicated that only two of these genes were found to be significantly induced after dissociation, though fold changes were small (*q* < 0.05, *Zfhx3* log2 fc = 0.39, *Sox4* log2 fc = 0.45, Supplementary Table 6). This implies that this list does not represent de novo induction of transcription factor expression as a result of cell extraction and sorting, but rather reflects enrichment in nociceptors.

## Discussion

We present the first application of magnetic cell sorting (MACS) to isolate and characterize nociceptor neurons from the adult DRG using RNA-sequencing. We find that MACS increases the purity of DRG sensory neuron cell body preparations from <10% to >95%, a purity that cannot be obtained through any other method. These MACS preparations are enriched for nociceptor neurons <30 μm in size expressing the voltage gated sodium channel, Na_v_1.8. Comparison of RNA-sequencing data in unsorted and nociceptor-enriched MACS-sorted preparations enables genome-wide resolution of the nociceptor transcriptome. We present novel biological insight into the ion channels, growth factor receptor isoforms and transcription factors present in these cells, and anticipate that both the novel sorting technique used and the data generated will be of great utility to researchers studying nociceptors in the context of pain, injury and regeneration.

The importance of attaining cell-type specificity in the context of high-throughput transcriptomic technologies (most often microarray and RNA-sequencing) is becoming increasingly clear (as reviewed in Okaty et al., 2011a; Cruz et al., 2013). Data generated from central nervous system (CNS) or peripheral nervous system (PNS) tissue, given the high level of heterogeneity, is subject to a number of limitations. The first is that any transcriptional change occurring in small populations of cells is likely to be underestimated if assessed in a composite tissue. Likewise, genes regulated in opposite directions in neighboring cell types will appear static in composite data. Similarly, transcripts which are differentially spliced in different cell types cannot be distinguished. Furthermore, following experimental manipulations such as injury, any change in the population of cells present in the tissue (such as infiltration of immune cells) complicates the attribution of any observed transcriptional change.

Obtaining cell-type specificity requires a means of identifying, and a means of isolating cells of interest. A number of features of adult neural tissue limit the applicability of the most common high throughput sorting technique, fluorescence-activated cell sorting (FACS), in PNS. The sensitivity of adult neuronal cell bodies to physical stress, the requirement for enzymatic disruption of tissue that may affect extracellular antibody epitopes, and the relatively small number of cells available make it difficult to recover sufficient RNA for high-throughput applications when flow sorting PNS tissue.

Another sorting technique that is suitable for transcriptome analysis of neural tissue is Translating Ribosome Affinity Purification (TRAP)-Seq. TRAP requires a transgenic mouse with a cell-type specific reporter allowing the labeling and isolation of ribosome-associated RNA within the cells of interest. One strength of the technique is that cell-type specific data can be pulled down from a composite tissue without having to separate constituent cell types. However, data generated using this technique does appear to be subject to a high degree of contamination from other cell types (Okaty et al., 2011b). Furthermore, TRAP is entirely dependent on the fidelity of the cell-type reporter used, and, in contrast to RNA-sequencing of whole-cell lysate, cannot detect non-coding RNA species.

Both FACS and TRAP require a transgenic reporter with high fidelity for the cell type of interest, and are not applicable to non-transgenic animals. In contrast, the MACS-sorting technique can generate pure neuronal preparations from standard laboratory animals with sufficient yield to carry out genome-wide sequencing.

Aside from transcriptomic studies, MACS cultures will also be extremely useful for *in vitro* study. Previously, preparing an enriched preparation of DRG neurons required extended culture (> 1 week) in the presence of a mitotic inhibitor to deplete contaminating non-neuronal cells (Lerch et al., 2012). This culture procedure is not widely used because of concerns about off-target effects of mitotic inhibitors on neurons (Wallace and Johnson, 1989), the likelihood that DRG neuron phenotype following prolonged culture will no longer resemble that of intact DRG (Buschmann et al., 1998) and the low purity of the culture it produces [74% neuronal (Lerch et al., 2012)]. For these reasons, most investigators simply use mixed cultures when studying DRG. However, for experiments focused on growth factor signaling, where contaminating non-neuronal cells are sensitive to and able to release growth factors, impurity of DRG culture is a significant limitation (Kalous et al., 2012). The ability to generate pure neuronal cultures will significantly simplify interpretation of *in vitro* experiments using dissociated DRG neurons.

Our study design allowed us to identify nociceptor-enriched genes by comparing global gene expression profiles of MACS-sorted small DRG neurons and dissociated whole ganglia. Further *in silico* analysis to compare nociceptor and cortical neuron transcriptomes revealed that the majority of nociceptor-enriched genes may also be PNS-specific, as these were found to be significantly upregulated compared to cortical neurons. These genes may therefore be useful drivers for nociceptor-specific Cre lines, or for PNS-specific drug targeting. Further investigation of other PNS neuronal subtypes, for example autonomic ganglia, would help to refine this list.

One limitation of our study is that we cannot definitively determine the source of genes that are expressed at lower levels in nociceptors compared to the whole DRG. This is because these transcripts could come from either large neurons or non-neuronal cells. However, we observed that known non-neuronal genes tend to have very low expression levels in MACS sorted cultures (FPKM < 0.5). We used this heuristic to interpret the alternate Ntrk2 isoform expression (Figure 4). Further work to isolate and transcriptionally profile all of the cell types of the DRG are required to verify the origin of depleted transcripts. However, in the absence of this information, the list of depleted genes may still be used to inform experiments. If researchers are interested in a particular receptor due to pharmacological efficacy, our differential expression data will help to identify whether results may be due to a direct action on nociceptors (i.e. if the receptor is enriched), or an indirect action (if the receptor is not present).

Many of the ion channels we identify in nociceptors (including *Kcnab1, Scn2*, and *Kcnc2*) have not previously been described in the PNS. Given the attractiveness of therapeutics targeting ion channels such as the sodium channel Na_v_1.7 (Eijkelkamp et al., 2012; Lee et al., 2014), these channels could constitute novel targets for the treatment of pain or itch. One interesting feature of the nociceptor transcriptome we observe is the relatively low enrichment of TRPV1 (log_2_ FC = 0.59) in the MACS-sorted preparation. This likely reflects the size-selection of the MACS method, which will exclude TRPV1+ A-fibers. This results in a relatively low enrichment for TRPV1 in the MACS-sorted preparation relative to unsorted DRG, as the latter contains these TRPV1+ A-fibers (Mitchell et al., 2010).

The nociceptor-enriched transcription factors we describe include a number with known roles in DRG morphogenesis and function. These include *Tlx3* and *Pou4f1*, which control the development of subsets of nociceptors (Lopes et al., 2012; Zou et al., 2012). Disrupting expression of nociceptor development-controlling transcription factors can be incredibly informative—for example, disruption of Runx1 reveals the genes uniquely expressed in cutaneous and deep-tissue innervating nociceptors (Yang et al., 2013), while Runx3 can be used to disrupt development of DRG proprioceptive neurons peripheral and central axonal projections (Inoue et al., 2002; Levanon et al., 2002).

Of the 70 genes with nucleic acid binding activity we identify in nociceptors, only 21 have a known function in DRG. Of these 21 previously characterized genes, many control neurite outgrowth and axonal regeneration following injury, including *Sox11*, *Pdlim1, Nfil3, Hivep3*, and *Smad1* (Jankowski et al., 2006; Wu et al., 2006; Macgillavry et al., 2009; Ohno et al., 2009; Zou et al., 2009). The remaining 49 uncharacterized genes include *Pou4f2* and *Atf5*, genes involved in the acute response to damage and axon outgrowth in the optic nerve, another popular model tissue for regeneration (Huang et al., 2014; Yasuda et al., 2014).

The finding of differential expression of the full length (FL) and truncated (T1) isoforms of the BDNF receptor TrkB suggests for the first time that only TrkB FL is present in nociceptors, with TrkB T1 expressed in non-neuronal cells of the DRG (Schwann cells or satellite glia). This advances previous studies in the CNS, where TrkB T1 was found in glia, but both forms were detected in neurons (Frisen et al., 1993; Armanini et al., 1995). The nociceptor transcriptome we present allows investigators to assess transcript-level differential expression for any gene of interest.

In summary, the DRG nociceptor preparation produced by MACS-sorting has many applications *in vitro* and in molecular studies. We have demonstrated how the nociceptor transcriptome, generated via RNA-sequencing of MACS-sorted DRG, can be mined to gain novel biological insight. Knowledge of the ion channels, splice isoforms and transcription factors enriched in nociceptors will be of great utility to investigators studying this tissue in the context of pain, somatosensation and itch. As sequencing data in a wider array of other tissues and cellular subtypes becomes available, it will deepen our understanding of the defining features of the nociceptor transcriptome.

## Author contributions

Data acquisition and analysis Matthew Thakur, Megan Crow, Natalie Richards, Gareth I. J. Davey, Emma Levine, Jayne H. Kelleher, Chibeza C. Agley, Stephen D. R. Harridge. Analysis of sequencing data/Bioinformatics Megan Crow, Franziska Denk. Drafting of manuscript Matthew Thakur, Megan Crow. Editing of manuscript Matthew Thakur, Megan Crow, Franziska Denk, Stephen B. McMahon.

### Conflict of interest statement

The authors declare that the research was conducted in the absence of any commercial or financial relationships that could be construed as a potential conflict of interest.
