# Supplementary material for: Defining the nociceptor transcriptome
Source: Front Mol Neurosci. 2014 Nov 11;7:87. doi: 10.3389/fnmol.2014.00087 (PMC4227287; doi:10.3389/fnmol.2014.00087)
Supplement: Supplementary file 7 [file Image1.PDF]

Supplementary Figure 1

Defining the nociceptor transcriptome

Matthew Thakur[1*+], Megan Crow[1*], Natalie Richards[1*], Gareth Davey[1], Emma Levine[1], Jayne H. Kelleher[1], Chibeza Agley[2], Franziska Denk[1], Stephen Harridge[2], Stephen B. McMahon[1]
[1] McMahon Neurorestoration Lab, King's College London, UK
[2] Centre of Human and Aerospace Physiological Sciences, King's College London, UK
*** These authors contributed equally**
[+]**Correspondence:** Dr Matthew Thakur, Wolfson CARD, Guy's Campus, King's College London, SE1 1UL. matthew.thakur@kcl.ac.uk

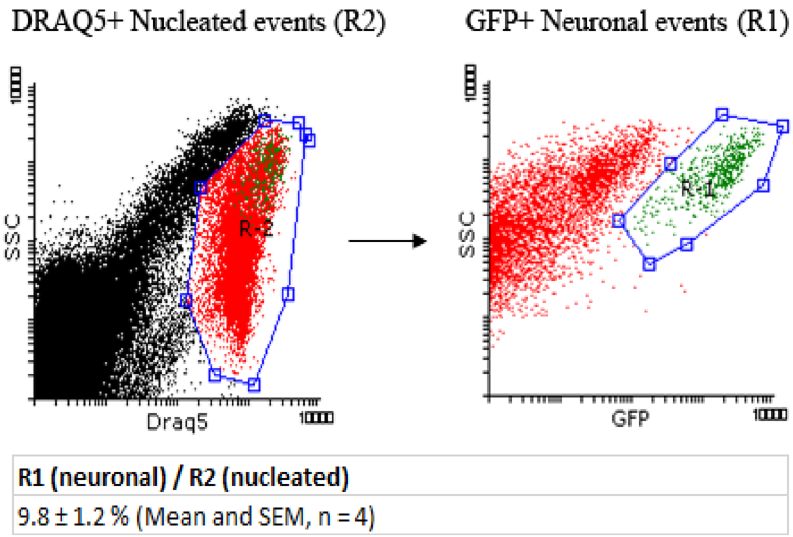

Supplementary Figure 1. Flow cytometry analysis of acutely dissociated, unsorted DRG neuron preparation. Nuclei are identified with DRAQ5, neurons from Adv-GFP animals express GFP. GFP+ neurons constitute an average of 9.8% of all nucleated cells in the preparation (SEM = 1.2, n = 4 independent experiments).
