# Supplementary material for: Defining the nociceptor transcriptome
Source: Front Mol Neurosci. 2014 Nov 11;7:87. doi: 10.3389/fnmol.2014.00087 (PMC4227287; doi:10.3389/fnmol.2014.00087)
Supplement: Supplementary file 8 [file Image2.PDF]

Supplementary Figure 2

Defining the nociceptor transcriptome

Matthew Thakur[1*+], Megan Crow[1]*, Natalie Richards[1]*, Gareth Davey[1], Emma Levine[1], Jayne H. Kelleher[1], Chibeza Agley[2], Franziska Denk[1], Stephen Harridge[2], Stephen B. McMahon[1]
[1] McMahon Neurorestoration Lab, King's College London, UK
[2] Centre of Human and Aerospace Physiological Sciences, King's College London, UK
*** These authors contributed equally**
[+]**Correspondence:** Dr Matthew Thakur, Wolfson CARD, Guy's Campus, King's College London, SE1 1UL. matthew.thakur@kcl.ac.uk

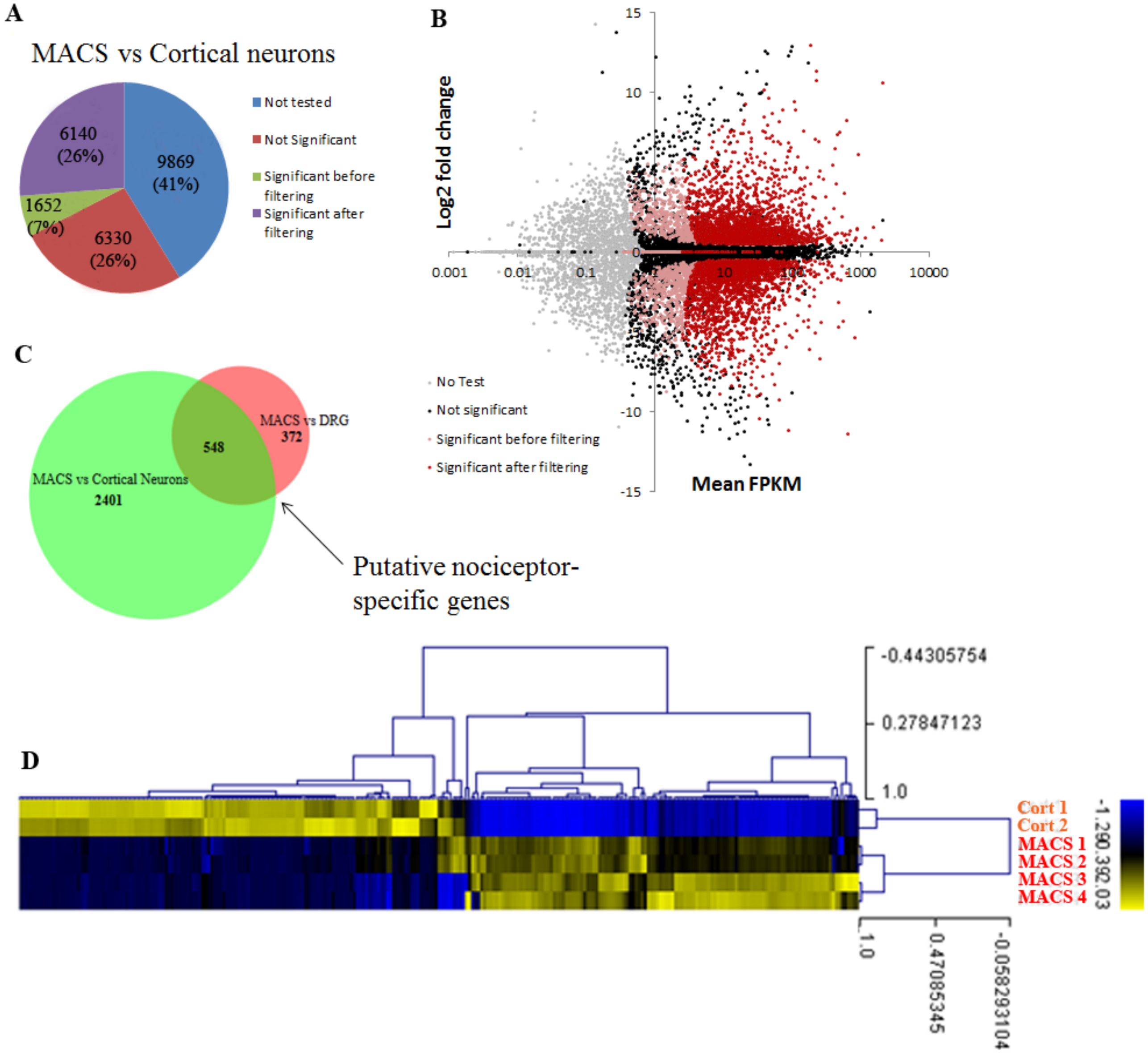

Supplementary Figure 2: Summary of *in silico* comparison between PNS nociceptor and CNS cortical neuron transcriptomes. A – Cuffdiff was used for differential expression testing between MACS-sorted DRG and cortical neuron samples. Of 14122 genes tested for differential expression, 6140 (26%) were significantly differentially expressed. B - Individual points represent genes. Numbers and percentages are those given in A. C - Focusing specifically on 920 genes enriched in small DRG neurons relative to unsorted DRG, 548 (68%) were also significantly enriched compared to cortical neurons. This suggests that these genes are not only nociceptor-enriched, but may in fact be nociceptor-specific. D - Heat map of the top 100 differentially regulated genes between cortical neurons and nociceptors. Genes are hierarchically clustered using average linkage clustering and expression is normalized by gene.
