# Supplementary material for: Defining the nociceptor transcriptome
Source: Front Mol Neurosci. 2014 Nov 11;7:87. doi: 10.3389/fnmol.2014.00087 (PMC4227287; doi:10.3389/fnmol.2014.00087)
Supplement: Supplementary file 9 [file Image3.PDF]

Supplementary Figure 3

Defining the nociceptor transcriptome

Matthew Thakur[1*+], Megan Crow[1*], Natalie Richards[1*], Gareth Davey[1], Emma Levine[1], Jayne H. Kelleher[1], Chibeza Agley[2], Franziska Denk[1], Stephen Harridge[2], Stephen B. McMahon[1]
[1] McMahon Neurorestoration Lab, King's College London, UK
[2] Centre of Human and Aerospace Physiological Sciences, King's College London, UK
*** These authors contributed equally**
[+]**Correspondence:** Dr Matthew Thakur, Wolfson CARD, Guy's Campus, King's College London, SE1 1UL. matthew.thakur@kcl.ac.uk

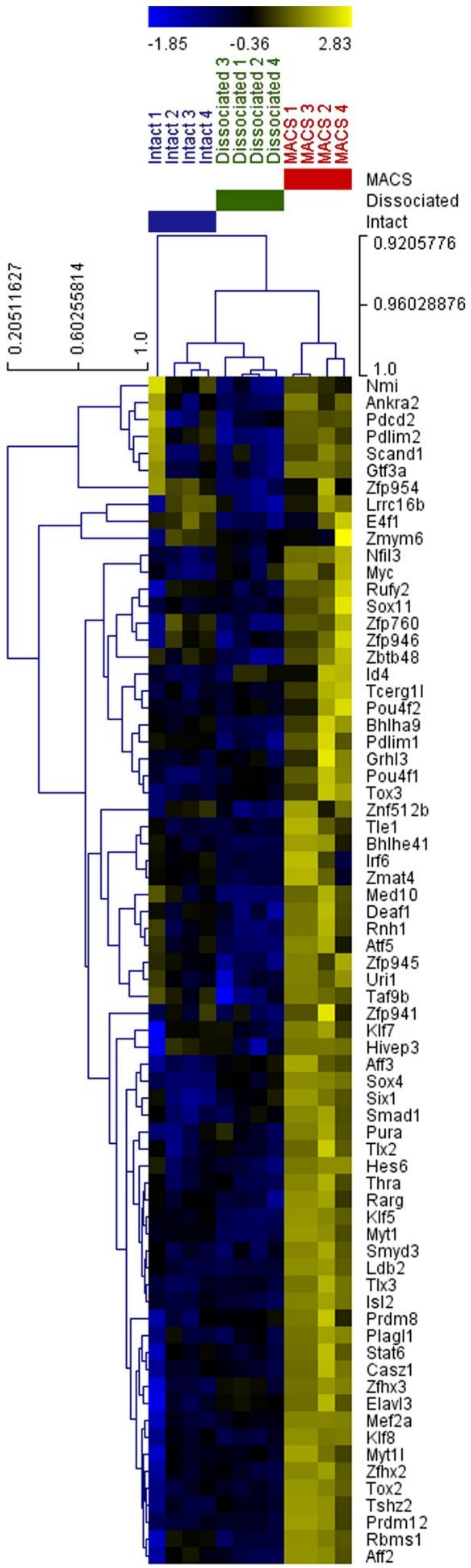

-1.85
-0.36
2.83
Intact 1
Intact 2
Intact 3
Intact 4
Dissociated 3
Dissociated 1
Dissociated 2
Dissociated 4
MACS 1
MACS 3
MACS 2
MACS 4
MACS
Dissociated
Intact
0.9205776
0.96028876
1.0
0.20511627
0.60255814
1.0
Nmi
Ankra2
Pdcd2
Pdlim2
Scand1
Gtf3a
Zfp954
Lrrc16b
E4f1
Zmym6
Nfil3
Myc
Rufy2
Sox11
Zfp760
Zfp946
Zbtb48
Id4
Tcerg1l
Pou4f2
Bhlha9
Pdlim1
Grhl3
Pou4f1
Tox3
Znf512b
Tle1
Bhlhe41
Irf6
Zmat4
Med10
Deaf1
Rnh1
Atf5
Zfp945
Uri1
Taf9b
Zfp941
Klf7
Hivep3
Aff3
Sox4
Six1
Smad1
Pura
Tlx2
Hes6
Thra
Rarg
Klf5
Myt1
Smyd3
Ldb2
Tlx3
Isl2
Prdm8
Plagl1
Stat6
Casz1
Zfhx3
Elavl3
Mef2a
Klf8
Myt1l
Zfhx2
Tox2
Tshz2
Prdm12
Rbms1
Aff2

Supplementary Figure 3. Heat map of 70 genes enriched in nociceptors that are associated with nucleic acid binding and transcription factor activity, identified through functional annotation analysis. Genes are hierarchically clustered using average linkage clustering and expression is normalized by gene.
