# Supplementary material for: Defining the nociceptor transcriptome
Source: Front Mol Neurosci. 2014 Nov 11;7:87. doi: 10.3389/fnmol.2014.00087 (PMC4227287; doi:10.3389/fnmol.2014.00087)
Supplement: Supplementary file 10 [file Image4.PDF]

# *Supplementary Figure 4*

# Defining the nociceptor transcriptome

**Matthew Thakur[1*+], Megan Crow[1*], Natalie Richards[1*], Gareth Davey[1], Emma Levine[1], Jayne H. Kelleher[1], Chibeza Agley[2], Franziska Denk[1], Stephen Harridge[2], Stephen B. McMahon[1]**
[1] McMahon Neurorestoration Lab, King's College London, UK
[2] Centre of Human and Aerospace Physiological Sciences, King's College London, UK
*** These authors contributed equally**
[+]**Correspondence:** Dr Matthew Thakur, Wolfson CARD, Guy's Campus, King's College London, SE1 1UL. matthew.thakur@kcl.ac.uk

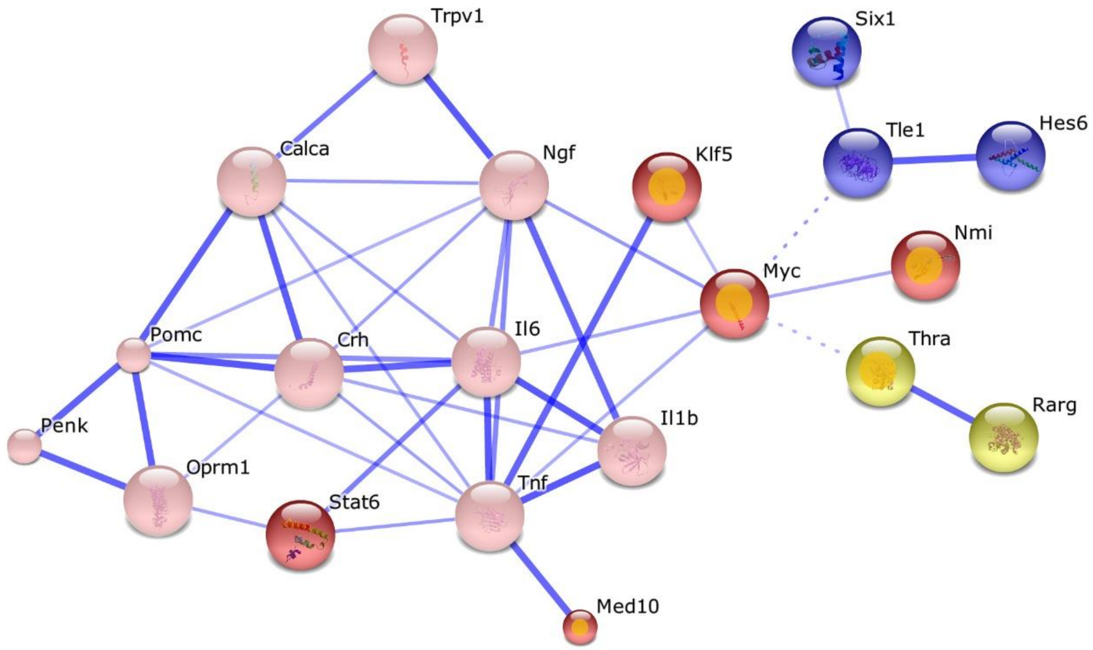


**Supplementary Figure 4.** Network analysis of nociceptor transcription factors and pain-associated genes. Pink nodes represent pain-associated genes from the pain interactome; orange spots indicate genes with no known pain association; thickness of the edge represents the confidence of the interaction. Thus *Klf5*, *Myc* and *Med10* are transcription factors previously uncharacterized in DRG but predicted to interact with key nodes of the pain interactome.
